# Detect and attribute the extreme maize yield losses based on spatio-temporal deep learning

**DOI:** 10.1016/j.fmre.2022.05.006

**Published:** 2022-05-16

**Authors:** Renhai Zhong, Yue Zhu, Xuhui Wang, Haifeng Li, Bin Wang, Fengqi You, Luis F. Rodríguez, Jingfeng Huang, K.C. Ting, Yibin Ying, Tao Lin

**Affiliations:** a College of Biosystems Engineering and Food Science, Zhejiang University, Hangzhou, Zhejiang 310058, China; b International Campus, Zhejiang University, Haining, Zhejiang 314400, China; c Sino-French Institute for Earth System Science, College of Urban and Environmental Sciences, Peking University, Beijing 100871, China; d School of Geosciences and Info-Physics, Central South University, South Lushan Road, Changsha 410000, China; e NSW Department of Primary Industries, Wagga Wagga Agricultural Institute, Pine Gully Road Wagga Wagga, NSW 2650, Australia; f Robert Frederick Smith School of Chemical and Biomolecular Engineering, Cornell University, Ithaca, NY 14853, USA; g Department of Agricultural and Biological Engineering, University of Illinois at Urbana-Champaign, Urbana, IL 61801, USA; h Institute of Applied Remote Sensing and Information Technology, Zhejiang University, Hangzhou, Zhejiang 310058, China; i Key Laboratory of On Site Processing Equipment for Agricultural Products, Ministry of Agriculture and Rural Affairs, Hangzhou, Zhejiang 310058, China; j Key Laboratory of Intelligent Equipment and Robotics for Agriculture of Zhejiang Province, Hangzhou, Zhejiang 310058, China

**Keywords:** Crop yield estimation, Deep Learning, Long short-term memory, Multi-task learning, Extreme yield loss, Attribution analysis

## Abstract

Providing accurate crop yield estimations at large spatial scales and understanding yield losses under extreme climate stress is an urgent challenge for sustaining global food security. While the data-driven deep learning approach has shown great capacity in predicting yield patterns, its capacity to detect and attribute the impacts of climatic extremes on yields remains unknown. In this study, we developed a deep neural network based multi-task learning framework to estimate variations of maize yield at the county level over the US Corn Belt from 2006 to 2018, with a special focus on the extreme yield loss in 2012. We found that our deep learning model hindcasted the yield variations with good accuracy for 2006-2018 (R^2^ = 0.81) and well reproduced the extreme yield anomalies in 2012 (R^2^ = 0.79). Further attribution analysis indicated that extreme heat stress was the major cause for yield loss, contributing to 72.5% of the yield loss, followed by anomalies of vapor pressure deficit (17.6%) and precipitation (10.8%). Our deep learning model was also able to estimate the accumulated impact of climatic factors on maize yield and identify that the silking phase was the most critical stage shaping the yield response to extreme climate stress in 2012. Our results provide a new framework of spatio-temporal deep learning to assess and attribute the crop yield response to climate variations in the data rich era.

## Graphical abstract

**Figure:**
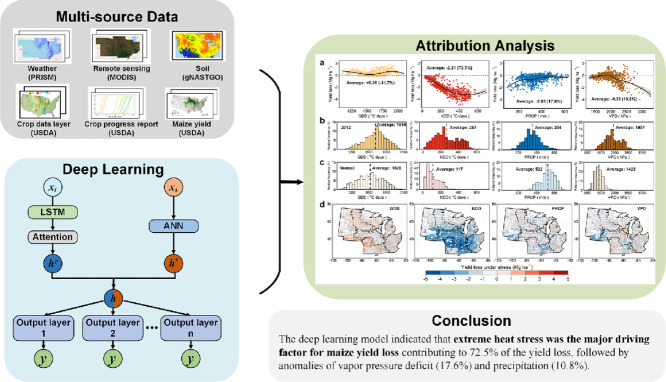

## 1 Introduction

Large-scale crop yield estimation is critical for understanding the dynamics of global food security. The food supply has become more vulnerable under global warming with increased frequencies of climate extremes [1,2]. Heat and drought stresses have been assessed as two driving factors for crop yield loss globally [3,4]. For instance, researchers found that drought and extreme heat significantly reduced national cereal production worldwide by 9%-10% during 1964-2007 [5]. It is critical to have an accurate and reliable crop yield model for understanding the relationships between yield and climate variability, particularly the impacts of heat and drought stresses.

Both statistical and process-based models have been applied to estimate yield loss in response to heat and drought stresses and their underlying mechanisms [2], [3], [4], [5], [6], [7], [8], [9], [10]. These studies, using different approaches, showed agreements on the qualitative impacts of extreme heat on crop yield. However, both statistical models and process-based models often underestimate the magnitude of yield loss in response to the extremes [7,8,11]. This has largely limited our capability to correctly estimate the yield variability under a more variable climate [12]. New approaches are urgently needed to better estimate the impact of heat and drought stresses on crop yield at large spatial scales to provide new insights into the potential impact on food security at regional to global scales.

Deep learning has shown good performance in extracting patterns from the ever-increasing stream of data, such as finding extreme climate patterns and detecting land-use and land cover change in the field of geoscience [13], [14], [15], [16]. The deep neural network approaches can discover intricate temporal or spatial relationships from high-dimensional data [17]. Recent studies also indicated these approaches, when they are well designed with appropriate input data streams, can be successful in estimating spatio-temporal variability of crop yield [18], [19], [20]. For example, the temporal changes and cumulative impacts of the environmental factors on crop yields have been captured through Long Short-Term Memory (LSTM) based models [[21], [22], [23], [24]]. These results suggest that the data-driven approaches would be more accurate than traditional approaches in capturing the nonlinear relationships between crop growth/development and the climate and soil factors. However, whether they could accurately estimate yield loss under the impacts of extreme climatic stresses is still an open question to investigate.

Another challenge for large-scale crop yield estimation is the spatial heterogeneity in environmental conditions and crop yield [25,26]. Crop yields vary substantially spatially as a result of various environmental conditions (e.g. climate and soil) and its associated seed hybrid selection by farmers. Consideration of the spatial variances in the relationship among the crop yield and environmental factors was evaluated by several spatial statistical studies [27,28], but it was not accounted for in most data driven models, which are critical for improved data driven modeling of the crop-environment relationship in quantifying the spatial variances and defining the regional boundaries.

Crop growth is complex temporal progress under the natural environment at large spatial scales. Understanding the influence of climatic factors across the growth period and detecting the key growth stages is critical for refining the understanding of the role of climate change and extreme events during crop production. The lack of interpretability is identified as a potential weakness of deep learning models [29]. The ‘black box’ characteristics inhibit the understanding of the causes and effects in the models. For example, how does the model predict the yield loss under extreme climate stresses? What are the key driving climatic factors and critical growth stages for the yield loss? How to assess the specific contributions of climatic factors on crop yield variations? These insights for large-scale crop production may be possible to be deciphered through explainable data-driven discoveries. Therefore, an explainable deep learning model could provide new data-driven insights for refining the understanding of the influence of climatic stresses in crop yield reduction and their spatio-temporal patterns at large spatial scales.

**Fig. 1:**
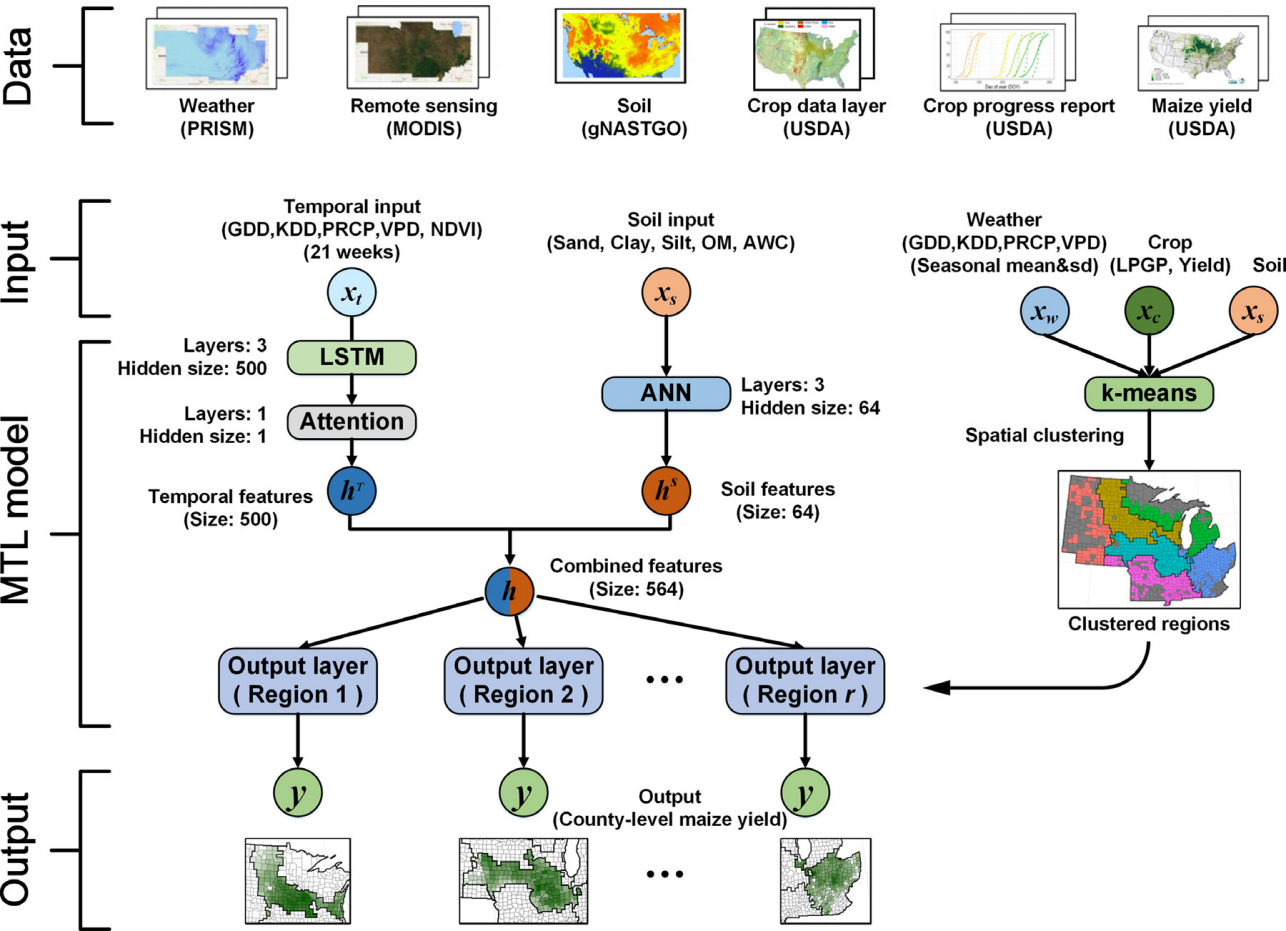
**The integrated workflow of deep neural network based multi-task learning model for county-level yield estimation**.

In this study, we established a deep learning framework by utilizing multi-source data (weather, soil, and remote sensing) for a better understanding of the crop yield response to climatic factors (Fig. 1). County-level maize yield in the US Corn Belt was used to demonstrate the model capability. We classified the US Corn Belt into several homogeneous maize production zones through an unsupervised clustering method and then developed a deep neural network based multi-task learning framework to extract the spatio-temporal patterns for yield estimation. Our focus was on understanding the responses of maize yield to normal and extreme climatic conditions at large spatial scales by conducting an attribution analysis based on the deep learning approach. This study aims to address the three research questions as follows:

• How to establish a deep learning workflow for large scale maize yield estimation considering the spatial variances of the environmental conditions in the US Corn Belt?
• Can the deep learning model well hindcast the spatio-temporal variations of maize yield in both normal and extreme climate conditions?
• What is the major climatic factor causing the 2012 extreme maize yield loss in the US Corn Belt from the perspective of deep learning?

## 2 Materials and methods

### 2.1 Datasets and data preprocessing

Our study focused on county-level rainfed maize yield in the 12 central and northern states in the US Corn Belt from 2006 to 2018 (Fig. S1). The 851 counties with 10,118 county-year records accounted for 76.4% of the total maize production of the US in the study period. The counties with yield records less than three years in each period were removed as they were not representative of the main maize production area. The county-level rainfed maize yield data and state-level Crop Progress Report (CPR) were obtained from USDA's National Agriculture Statistics Service [30]. The growing season was defined as the 21-week period, from week 18 to 38 of a calendar year according to the CPR.

We consolidated weather data, remote sensing data, and soil data to estimate the county-level maize yield of each year. The daily weather data were aggregated at the county-level by mean value based on the Parameter-elevation Relationships on Independent Slopes Model (PRISM) dataset with a 4-km resolution [31]. The primary climatic variables included daily minimum, maximum, and mean temperature; daily total precipitation; and daily minimum and maximum vapor pressure deficit. Daily climatic factors were calculated based on these variables, including Growing degree days (GDD), Killing Degree Days (KDD), Precipitation (PRCP), and Vapor Pressure Deficit (VPD). We used Normalized Difference Vegetation Index (NDVI) to characterize the maize growth condition. NDVI was derived from NASA's MODIS product MCD43A4 at 500 m resolution [32]. Non-maize pixels were masked based on the Crop Data Layer (CDL) from USDA [33]. The masked NDVI were aggregated as county-level by using the median value to minimize the errors caused by the extreme value. The soil properties, including Available Water Capacity (AWC), Organic Matter (OM), Sand Content, Clay Content, and Silt Content, were obtained from the Gridded National Soil Survey Geographic Database (gNATSGO) [34]. The raw data were at 10 m spatial resolution and aggregated at county-level by the mean value. All weather and remote sensing data were processed through the Google Earth Engine (GEE) platform [35] and the soil data was processed through ArcGIS 10.5 with the Soil Data Development Toolbox. For spatial visualization, all the maps were produced based on a standard map GS(2021)5456.

### 2.2 Framework for maize yield estimation

We developed an integrated workflow for county-level crop yield estimation by incorporating spatial clustering, deep neural network based feature extraction, and multi-task learning (Fig. 1). The model used multi-task learning to achieve region-specific pattern recognition so that we abbreviated the model as MTL. The environment (weather and soil) and yield information were used as the input to the spatial clustering module to quantify the spatial variance and cluster the Corn Belt in the US into several homogeneous regions. The feature extraction module extracted the temporal and soil patterns separately according to the specific input and network structure. For temporal dynamical features, we used the Attention-based LSTM (ALSTM) network to learn the temporal pattern from the time-series inputs of climatic factors and vegetation index derived from remote sensing data. For soil input, an Artificial Neural Network (ANN) was used to extract the feature from the soil data. Then, the temporal and soil features were fused and combined with the clustering results for region-specific yield estimation using multi-task learning. The output is the county-level maize yield at the end of growing season.

#### 2.2.1 Spatial clustering module

We applied the k-means clustering method to classify the entire US Corn Belt into several relatively homogenous maize production regions [36]. Historical average and variability of the environmental factors and maize yield were considered as inputs for spatial clustering. The factors were grouped into three categories: crop-related information, weather data, and soil properties. Crop-related factors included the average yield of maize during two periods (2006-2011 and 2013-2018) to reflect the spatial distribution of the maize production, and the length of the potential growth period (LPGP) according to the temperature conditions. The yield in 2012 was not considered due to the severe heat and drought stresses that caused the abnormal yield reduction. The LPGP was defined as the period between the first spring planting date (> 13 °C) and the first autumn frost date (< 0 °C). Climatic factors, including GDD, KDD, PRCP, and VPD, represented the weather conditions during the potential growth period.

Based on the crop-related information, weather data, and soil properties, six maize production regions were preferred based on the comparisons of the estimation accuracy by MTL models from a range of 2, 4, 6, and 8 clusters. Any isolated county was merged into a connected region to ensure the integrity of each classified spatial region, and all counties belonging to the same spatial region must be spatially connected. In addition, we applied t-distributed stochastic neighbor embedding (t-SNE) [37] to qualitatively measure the separability of the counties across different clustered regions. The spatial clustering process used here can be applied to other crops and in other regions.

#### 2.2.2 Feature extraction module

A feature extraction module was developed to process the spatial and temporal inputs, explicitly considering the spatial and temporal characteristics of multi-source data in our study. For the temporal dynamical input, an ALSTM network was developed to capture the temporal features [22]. The temporal dynamical input was a time series of weekly climatic variables (GDD, KDD, PRCP, and VPD) and NDVI. The hidden feature captured by the ALSTM network was defined as temporal feature and its dimension was set as 500. The length of the time series was set as 21 timesteps because maize growing season at Corn Belt has an average of 21 weeks based on the CPR. The ALSTM network consisted of a three-layer LSTM network and a one-layer attention network. The LSTM network was used to transport and store information selectively from the time-series input for temporal pattern extraction. The multiple LSTM layers enabled the LSTM network to generate hierarchical features from layer to layer. The attention network was one single-layer fully-connected neural network to generate different attention weights to reflect the importance of hidden features to maize yield in the corresponding period. The final temporal feature extracted by the ALSTM network was the combination of attention weights of the attention network and hidden features of the LSTM network. For the soil input (five soil properties), an ANN was developed for the feature extraction as the soil properties were considered temporally static with variances across geographical locations. We applied a three-layer ANN to capture the patterns of the soil conditions. The size of the three ANN layers was set as 64. Then, the temporal and soil features were concatenated for the subsequent yield estimation.

#### 2.2.3 Yield estimation module by multi-task learning

Based on the clustering results, multi-task learning was applied to develop output layers specific for each region by considering the spatial variances. Multi-task learning was to treat each region as an individual task to adjust the regional environmental conditions. The shared network that was concatenated by the ALSTM and ANN networks was to extract the general climatic and soil features across different regions, whereas the region-specific output layers by multi-task learning were to optimize specific weights based on local characteristics for minimizing the yield estimation losses. The output was the county-level maize yield at the end of the growing season. The loss function used was the Mean Squared Error (MSE) between the estimated and actual maize yield. This yield estimation module by multi-task learning enables the model to learn both specific patterns in each region and general patterns in the whole study area for better performance at large spatial scales. The details of the calculation process are described in the supplementary information.

### 2.3 Model performance evaluation

We developed three models as baseline models: least absolute shrinkage and selection operator (LASSO), random forest (RF), and ALSTM model. All the hyperparameters for the three models were selected based on the evaluation of cross-validation. LASSO regression model is composed of weekly inputs of four climatic factors (GDD, KDD, PRCP, VPD) and the three interaction terms among the stress-related climatic factors (KDD*PRCP, KDD*VPD, PRCP*VPD). The λ value is selected as 0.005 to avoid overfitting. For RF, the tree number was set to 5,000, and 37 of 110 input variables (5 temporal climatic input×21 timesteps +5 soil input) are randomly taken as candidates at each split. We set the maximum number of nodes for each tree as 300. The glmnet and randomForest R package were used to train LASSO and RF, respectively. The structure of the ALSTM model was similar to that of the MTL model. The difference is that the ALSTM model estimates the yield by only one output layer without the multi-task learning module.

MTL and three baseline models were trained and evaluated under the same scenario. We evaluated the model performance through the leave-one-year-out cross-validation based on 10,118 records of 851 counties in the US Corn Belt from 2006 to 2018. In the leave-one-year-out cross-validation, we selected samples in one of the 13 years as the test set in turn and left the remaining 12-year samples as the training set to evaluate the model performance in estimating the interannual variability of maize yield. The Root Mean Squared Error (RMSE), Coefficient of determination (R^2^), and Mean Error (ME) between the estimated and actual yield were used as the evaluation criteria. Furthermore, we focused on the model performance in both normal and extreme climatic conditions.

### 2.4 Attribution analysis of the 2012 maize yield loss

We quantified the contributions of each climatic factor to understand the major driving factors of the 2012 maize yield loss. The attribution analysis was based on comparing the yield differences between estimated yield under normal and stressful climatic input. The comparisons were based on the models (MTL, RF, and LASSO) tested in the year of 2012. We first calculated the historical average weekly inputs of the four climatic factors (GDD, KDD, PRCP, VPD) at county-level except 2012 to represent the normal climatic conditions and identified an estimated yield at normal condition (Eq. 1). We replaced the normal input with the 2012 stressful input at a 3-week interval cumulatively from planting to estimate the yield response to each climatic stress till the corresponding growth stage (Eq. 2). The difference between the estimated yield under normal and stressful conditions was defined as the yield reduction caused by each climatic factor till the corresponding growth stage (Eq. 3).

(1) $$Y_{c}^{normal}=f\left(x_{c}^{normal}\right)$$

(2) $$Y_{c,p}^{i}=f\left(x_{c,p}^{i}\right)$$

(3) $$\Delta Y_{c,p}^{i}=Y_{c,p}^{i}-Y_{c}^{normal}$$

where $x_{c}^{normal}$ was the historical average input in county *c*, the function f represented the models, $Y_{c}^{normal}$ was the estimated normal yield, *i* was the index of the climatic factors (GDD, KDD, PRCP, VPD), *p* represented the stages at a 3-week interval from planting to mature with a range from one to seven, $x_{c,p}^{i}$ represented the input in county *c* with corresponding climatic factor *i* that suffered the stress till the stage *p* in 2012, and $Y_{c,p}^{i}$ was the estimated yield under corresponding stress. For example, $Y_{1,3}^{KDD}$ represents the estimated yield in county 1 when the maize suffered heat stress of 2012 represented by KDD in a period of three stages (nine weeks) from planting but with climatic factors in later weeks till mature as normal values. The yield loss ($\Delta Y_{c,p}^{i}$) was calculated as the difference between $Y_{c,p}^{i}$ and $Y_{c}^{normal}$.

In addition, we calculated the average 2012 yield loss in the US Corn Belt by the mean of differences between the linear trend yield and real yield for each county (Eq. 4). The relative contribution of maize yield loss by each climatic factor was calculated (Eq. 5).

(4) $$\bar{\Delta Y}=\frac{\sum_{c=1}^{n}\left(Y_{c}^{2012}-Y_{c}^{2012t}\right)}{n}$$

(5) $$R^{i}=\frac{\sum_{c=1}^{n}\left(\Delta Y_{c,gp}^{i}\right)}{\bar{\Delta Y}}$$

where $Y_{c}^{2012}$ was the 2012 yield in county *c*, $Y_{c}^{2012t}$ was the linear trend yield of county c in 2012 that represented the ideal yield under normal climatic conditions, $\bar{\Delta Y}$ was the average county-level yield loss in the US Corn Belt,$\;\Delta Y_{c,gp}^{i}$ represented the yield loss caused by the climatic factor *i* during the whole growth period, and $R^{i}$ was the relative contribution of the estimated yield loss caused by climatic factor *i* to the average 2012 yield loss.

## 3 Results and discussion

### 3.1 Spatial variances of yield and environmental factors

**Fig. 2:**
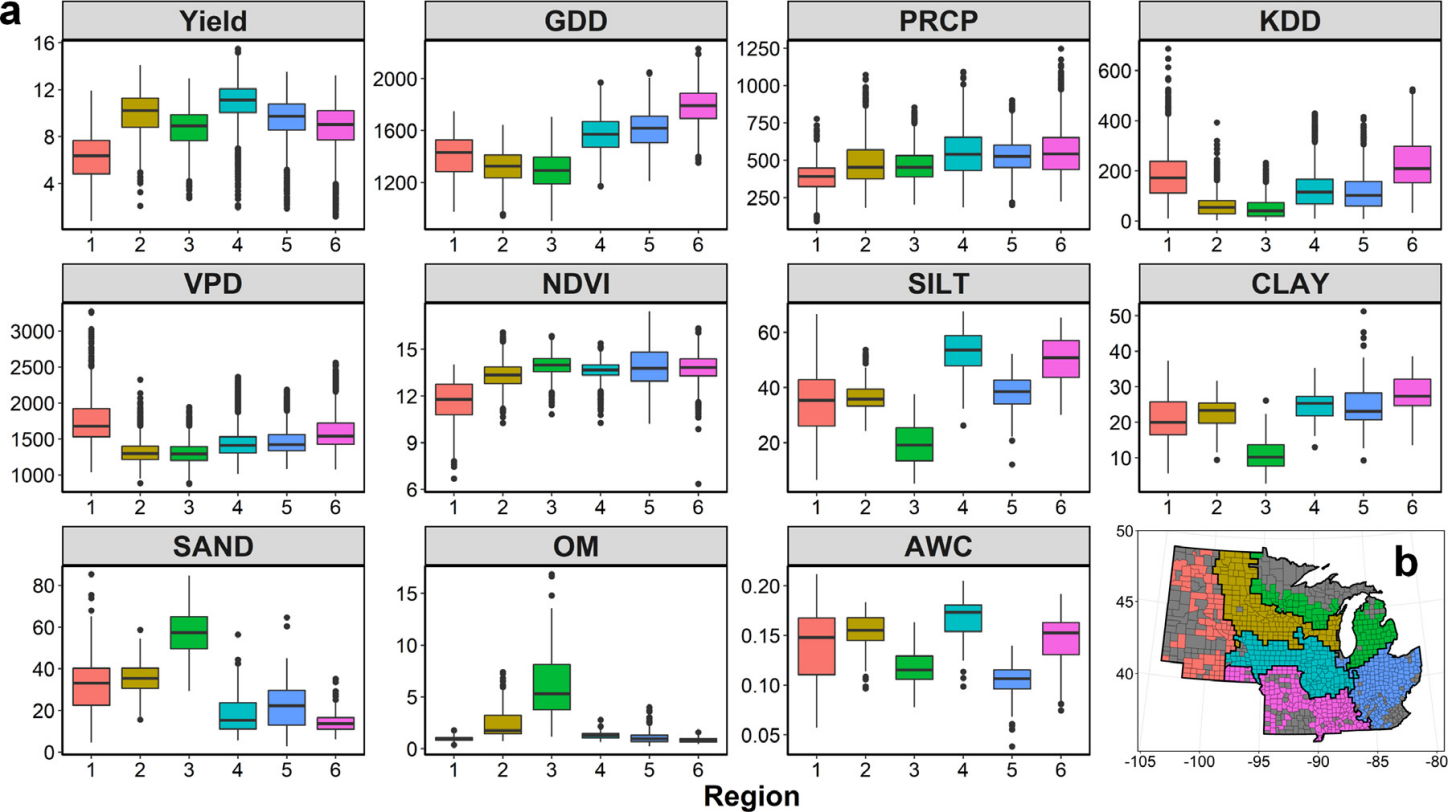
**Spatial distribution of the clustered regions and their maize growth conditions**. (a) Boxplots of the county-level maize yield and growing season accumulated input variables highlight the spatial variances across regions in the US Corn Belt; (b) the county-level map of the US Corn Belt is separated into six regions based on k-means clustering, where the same color surrounded by the black boundary is defined as one relative homogenous region with a similar maize growth condition classified based on k-means clustering and counties in grey color are with no or insufficient maize records. Abbreviations: GDD, Growing degree days; PRCP, Precipitation; KDD, Killing degree days; VPD, Vapor pressure deficit; NDVI, Normalized difference vegetation index; SILT, Silt content; CLAY, Clay content; SAND, Sand content; OM, Organic matter; AWC, Available water capacity.

The maize productivity showed considerable spatial variances across the clustered regions (Fig. 2a). We classified the Corn Belt under study into six relatively homogenous regions based on the k-means clustering method (Fig. 2b). The regional variability of historical maize yields could be large. Specifically, the region 4 (central Iowa and northern Illinois) remained the most productive region with the highest average maize yield (10.93 Mg ha^−1^). Conversely, region 1 (western North Dakota, South Dakota, and Nebraska) had the lowest average rainfed maize yield (6.18 Mg ha^−1^) (Fig. 2a). The yield difference illustrated that considering the spatial variability was essential for maize yield estimation over large scales.

The spatial variance in corresponding environmental factors in each subregion was the major driver of such productivity difference (Fig. 2a). The climatic factors in each growth period showed a consistent relative pattern in each region, which might contribute to the yield variance across different regions. For example, region 4 had the ideal maize growth environment with sufficient GDD and PRCP and less KDD and VPD. For region 1, the insufficient GDD accompanied by high KDD and VPD resulted in a low maize yield. Also, the spatial distribution of the soil conditions was an important driver for the yield variances. The soil in region 4 had the highest percentage of Silt and the highest AWC to maintain nutrient and moisture. Furthermore, the result of t-SNE showed that the samples in each region were well grouped (Fig. S2), which indicated that the clustered regions were well defined based on their complex high-dimension data. The differences in yield and environments across different regions implied that our spatial clustering approach captured the spatial pattern from the yield variability and its major driving environmental factors. It should be noted that the spatial variances were quantified by a data-driven approach, so that the classifying framework can be adjusted based on the updated data.

### 3.2 The MTL model performed well in both normal and extreme climatic conditions

**Fig. 3:**
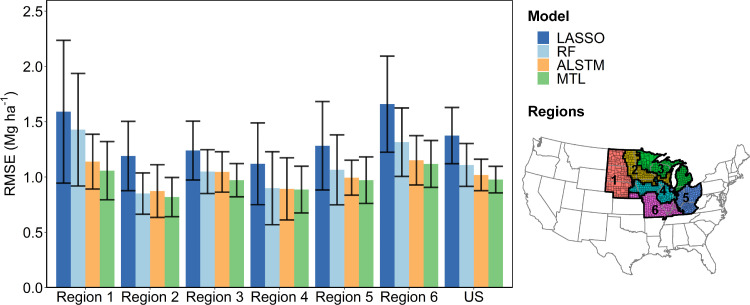
**Performance of four models (LASSO, RF, ALSTM, and MTL) for county-level maize yield estimation in each region and the whole US Corn Belt**. Bars and error bars indicate the means ± s.d. of the annual RMSE values in the leave-one-year-out cross-validation that models were iteratively trained using 12 years data and tested using the remaining one-year data during the period of 2006-2018. ALSTM and MTL represent the LSTM-based deep learning models without and with multi-task learning, respectively. The map in the lower right corner shows the six geographic regions based on unsupervised spatial clustering (details can be found in methods). The MTL model conducted the yield estimation in each respective region as one of the six specific tasks via multi-task learning. The significance levels of performance differences among the models are provided in table S1.

All models’ performances varied spatially, which might result from different environmental conditions and yield responses across the six regions (Fig. 3). Among the models, the MTL model captured 81% of the county-level yield variations and provided the highest estimation accuracy, with an average overall RMSE of 0.98 Mg ha^−1^. The MTL model achieved the lowest estimation RMSEs in all regions, with regional RMSEs ranged from 0.82 to 1.12 Mg ha^−1^. Both LSTM-based deep learning models, ALSTM and MTL, showed higher estimation accuracies with lower fluctuations than LASSO and RF. The results indicated that the LSTM-based deep learning models provided a promising tool for capturing the complex spatio-temporal relationships between crop yield and environmental factors. The MTL model yielded a more accurate estimation when compared to the ALSTM model, owing to the region-specific output layers that adjusted the spatial variances across different regions, illustrating the necessity to consider region clustering. We also compared the models’ performance at each county by calculating the county-level RMSEs (Fig. S3) and the MTL model showed lower RMSE distribution compared with the other three models. The results implied that the deep learning model was a promising alternative for better capturing the crop-environment relationships with the assistance of increasing varieties and improving quality of the observation data.

**Fig. 4:**
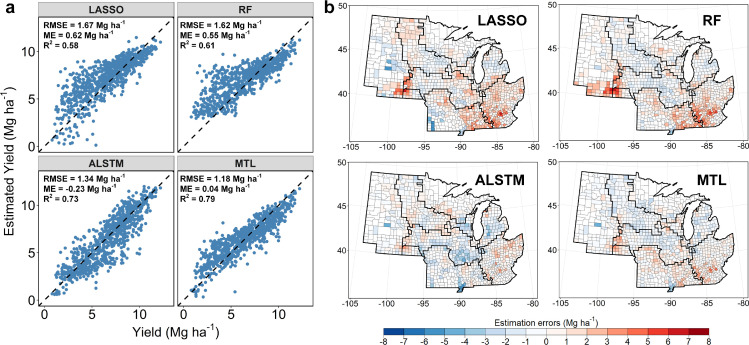
**Model performance comparison under extreme heat and drought conditions in the year of 2012.** (a) Scatterplots of county-level yield vs. estimated yield. (b) Maps of the estimation error between the estimated and actual yield at county-level. ME represents the mean error, and RMSE represents the root mean squared error.

The good performance of our deep learning models in hindcasting the spatio-temporal variations of the maize yield also implies that, unlike traditional modeling approaches which often underestimate extreme yield loss [8,9], deep learning models could well reproduce extreme yield loss, such as that in 2012, when US Corn Belt was largely affected by the extreme heat and drought conditions (Fig. 4). The deep learning models showed better performance for the yield loss estimation than LASSO and RF. The MTL model achieved the lowest overall RMSE of 1.18 Mg ha^−1^ and mean error of 0.04 Mg ha^−1^. The LASSO and RF models tended to underestimate the yield loss by extreme climatic stresses, with positive mean errors of 0.62 and 0.55 Mg ha^−1^, respectively (Fig. 4a). Both MTL and ALSTM models showed noticeable improvements to LASSO and RF models in regions 1, 5, and 6, where the maize yields suffered most based on the historical data. The average losses in the three regions ranged from -2.7 to -5.2 Mg ha^−1^ (Fig. S4). The results demonstrated that LSTM-based recurrent neural network could better capture the extreme climatic stress on crop yields. Furthermore, given the spatial variance of climatic stress and soil conditions, the MTL model can further improve the performance through multi-task learning of local patterns. The improvement could be explained by the local pattern captured by the multi-task learning that enabled the model to better estimate the impact of climatic stresses in each region. The results demonstrated that the MTL model could well reproduce the yield loss under extreme climate conditions.

### 3.3 KDD was the major driving factor of the 2012 yield loss

**Fig. 5:**
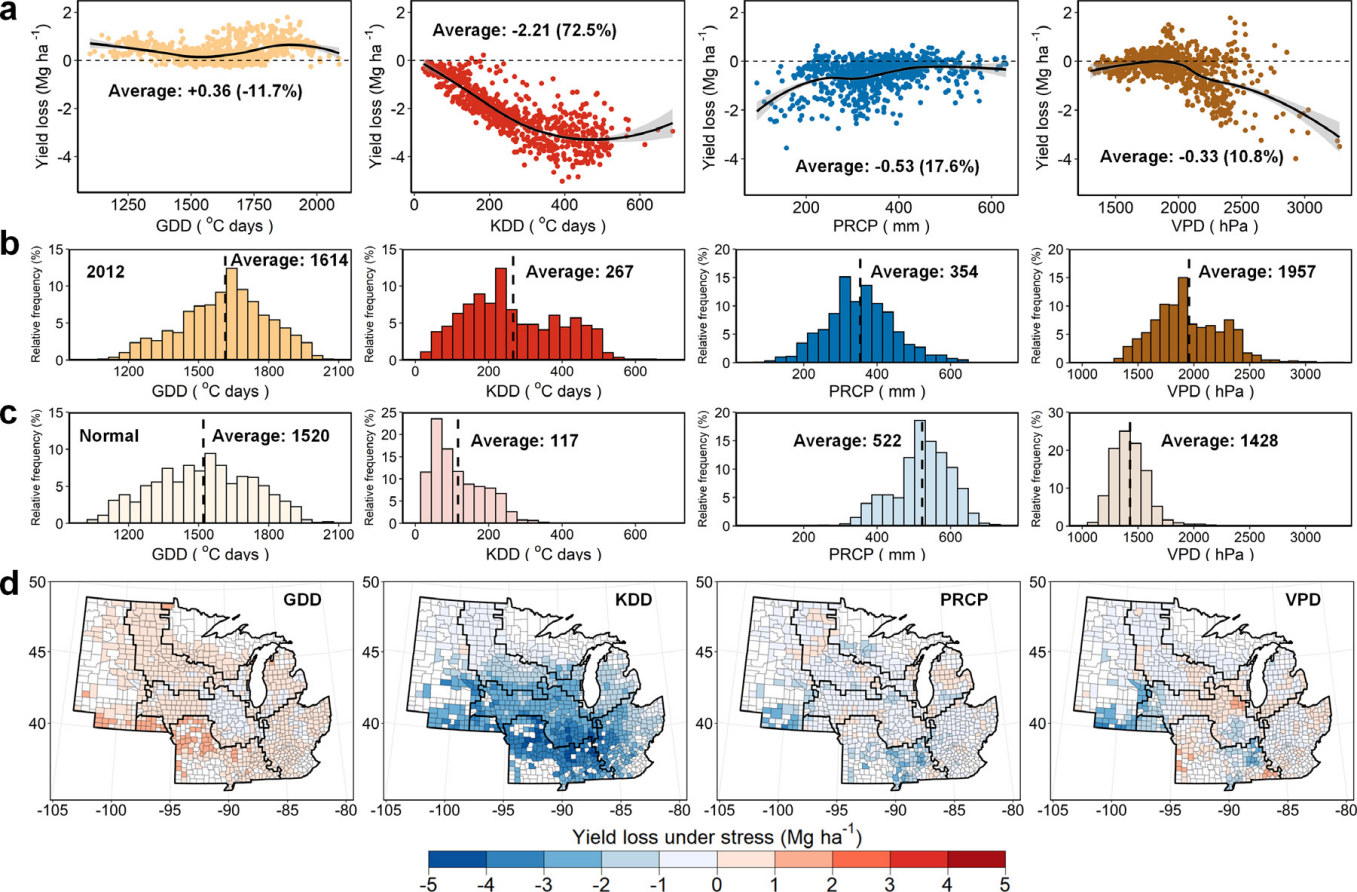
**Relationships between yield loss and climatic factors and the spatial patterns of yield loss.** (a) The scatterplots between the estimated yield reduction and the corresponding stress in 2012. The yield loss represents the estimated yield reduction caused by the responding climatic stress of 2012 compared to the yield in normal years. The percentage represents the relative contribution of the corresponding factor to the average yield loss in 2012. (b) Histograms of the relative frequency of the seasonal cumulative climatic factors in 2012. (c) Histograms of the relative frequency of the seasonal cumulative climatic factors in normal years. The vertical black lines represent the average values of the seasonal climatic factors. (d) The county-level maps of estimated yield loss caused by the corresponding climatic stresses in 2012.

The attribution analysis showed that the KDD was the major driver of the 2012 yield loss, which accounted for 72.5% of the average yield loss (Fig. 5a), whereas anomalies in PRCP and VPD also led to yield losses but with much smaller relative contribution (17.6% and 10.8%, respectively). GDD, as an indicator of the accumulated heat for crop growth, showed an overall positive impact on the yield with 0.36 Mg ha^−1^ yield increase on average. However, the slight positive impact of GDD was much lower than the negative influence of extreme heat. The increased magnitude of heat stress in 2012 was the largest, with an average cumulative KDD during the growth period being 2.3 times the historical average level (Fig. 5b and c). In 2012, the seasonal PRCP decreased by 32.2%, and VPD increased by 37.0%. The yield reduction was strongly associated with KDD, which agreed with previous empirical analyses [6,38]. All factors showed non-linear impacts on the yield, with PRCP lower than 300 mm or VPD above 2300 hPa as the apparent threshold where yield reduction becomes obvious. The results imply that the high sensitivity of yield to water stress usually happens in drier regions of the Corn Belt. The non-linear patterns were consistent with the knowledge that the damage on crop usually occurred when the stress was above a certain threshold [39,40].

Spatially, the yield loss caused by the stress of KDD was widely spread, with 57% of counties having more than one-fifth of yield losses (Fig. 5d). Whereas the low PRCP and high VPD only affected 5% and 6% counties with more than one-fifth of yield losses, and these counties were concentrated in the southern part of region 1. The estimated yield loss showed consistent spatial patterns with the USDA report (Fig. S4). Region 6, the southernmost area, suffered the highest yield loss due to the extreme heat. Whereas the yield losses were the lowest in regions 2 and 3, as they were located in the northern areas that experienced less heat stress. The results demonstrated that the MTL model could not only identify the major drivers of the yield loss, but also well represent the spatial patterns of the climatic stress impact. The spatio-temporal deep learning model showed the possibility to provide insights for a better understanding of the spatial patterns of crop-environment relationships at the regional to global scales.

**Fig. 6:**
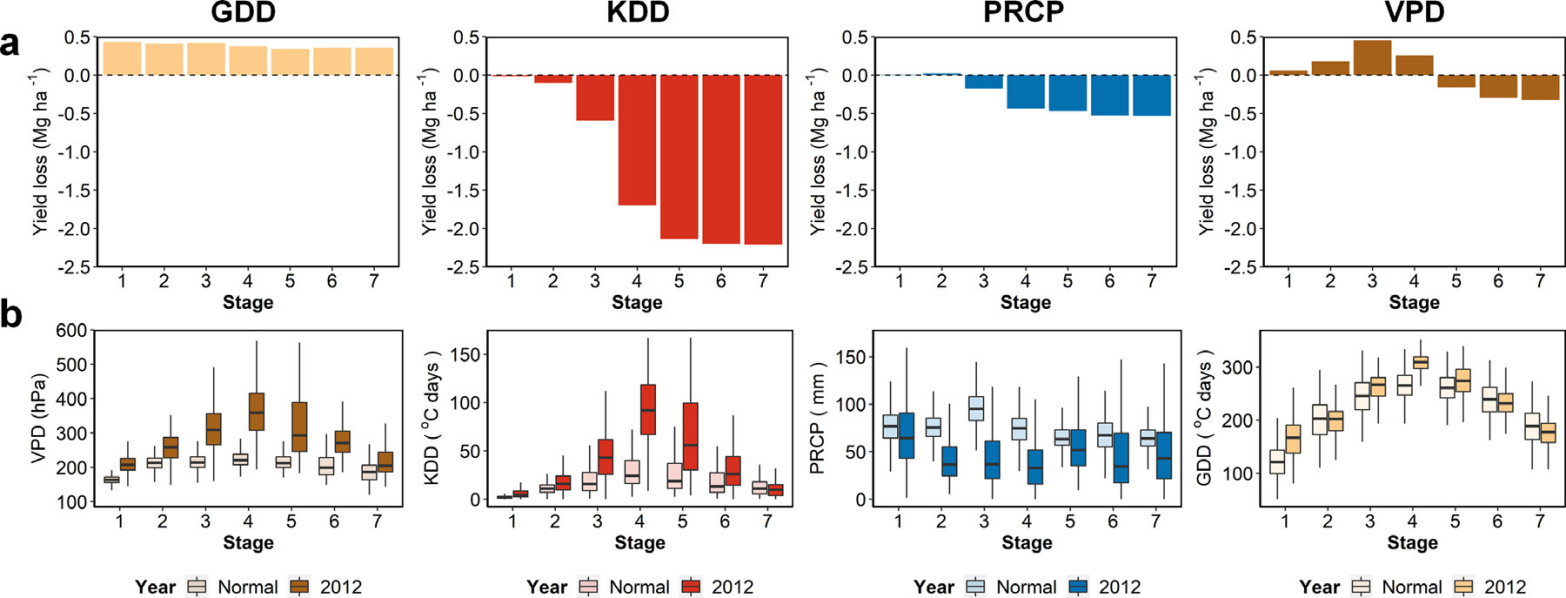
**The estimated yield loss and corresponding climatic conditions at each stage during the maize growth period in 2012.** The horizontal axes represent the seven consecutive three-week intervals throughout the whole growth period. (a) The estimated average yield losses as the maize suffered the climatic stresses in 2012 from planting to the corresponding stage. (b) Boxplots of the climatic conditions at each stage in the normal years and 2012.

The influence of the climate stresses accumulated over the maize growth period, with higher impacts during certain key stages (Fig. 6a). The negative influence of KDD on maize yield was most severe at stage four, when it was during the silking phase. The KDD in that stage also showed the largest difference between the normal years and 2012 (Fig. 6b). Detailed regional results showed a consistent pattern that silking phase was the most vulnerable stage in all regions (Fig. S5). The key stage found through deep learning was consistent with the existing literature, including simulated studies based on process-based and empirical models and agronomy studies [41,42]. Besides the vulnerability under key stages was represented, we found that the model was able to simulate the cumulative influence of climatic stress in each phase on the final yield loss. The high VPD in the early period showed a positive impact, which could be explained by the accelerated growth and higher root-specific capacity for nutrient uptake under that condition [43,44]. However, the continuous high VPD affected the silking and grainfilling, finally resulting in a yield reduction. The cumulative yield reduction showed less variations during the periods close to planting and maturity when the maize was less sensitive to the environment. In addition, the cumulative influences of GDD, KDD, and PRCP showed similar trends across regions, but with magnitudes varied across regions because of the variances of the regional stress levels. These temporal patterns were captured through the LSTM-based structure that enabled the model to selectively extract and store information from the time-series data during the crop growth. The results indicate that the deep learning approach provides data-driven insights on how climatic factors affect the crop growth temporally, especially when the crop is under key growth phases.

The observed mechanisms between crop growth and climatic stresses varied among different models. The MTL model found that KDD was the most important factor that reduced the maize yield of the US Corn Belt in 2012. The RF model, however, quantified the VPD as the most influential factor that accounted for 28.8% of the 2012 yield loss (Fig. S6). For the LASSO model, the yield loss was also mainly caused by the KDD, but only accounted for 42.7% (Fig. S7). The underestimation of yield loss by RF and LASSO might be explained by the incorrect mechanism captured by the two models. They both obviously underestimated the influence of KDD, particularly for the RF model. The unregular temporal yield loss estimated by LASSO also indicated that LASSO was unable to capture the cumulative effect of crop growth. Previous studies have also shown the climatic drivers of yield loss were divergent. For example, related studies observed the crop-environment relationships that higher impact of KDD than precipitation [3,4,6], whereas VPD was the most influential factor in other simulations [2,[45], [46], [47]]. In addition to the environmental factors, maize genotype and agronomic management are other essential factors for yield estimation. Further improvement could be done by integrating these factors into the deep learning model to provide a new alternative to reveal the underlying mechanism for decision support in agriculture, such as hybrid performance evaluations, adaptation strategies, and climate change impact projections.

## 4 Conclusion

We have developed a spatio-temporal deep learning model for county-level crop yield estimation, in both normal and extreme climate conditions. Results showed that the model performed well in both climate conditions and could robustly represent the extreme yield loss in 2012. The deep learning model was found to be able to provide a promising alternative for large-scale crop yield estimation, which overcomes the large structure and parameter uncertainties of process-based models and the quasi-linear structure of the statistical models. We expect that the robust capability in yield estimation for extreme climatic conditions can be applied for future yield projections under different climate change scenarios. In addition, the MTL model is a data-driven and flexible framework and can be extended to other crops in different regions with proper adjustments. For example, it can be further transferred to other regions through transfer learning approaches (e.g. fine-tune and domain adaptation) to provide systematic insights for yield estimates at local to global scales.

We proposed an attribution analysis approach to quantify the impacts of individual climatic factors on extreme maize yield loss based on the deep learning models. Our attribution analysis has identified KDD being the major driver of the unusual yield loss in the US Corn Belt in 2012. This conclusion was further supported by the less well performance of more traditional machine learning models like RF and LASSO, who underestimated the impacts of KDD. In addition, the detailed seasonal analysis further found that silking was the most sensitive stage for maize yield loss responding to heat stress. We noted that the low probability of these high-impact events has limited our capacity to further demonstrate the robustness of the impacts of climatic stresses, which calls future studies to more thoroughly investigate both the temporal and spatial thresholds of climatic stresses.

To our knowledge, our study is the first such effort of using the deep learning models to quantify the impacts of individual climatic factors on extreme maize yield loss. By doing so, we move forward from treating the deep learning models as “black box” prediction tools to interpretable tools that could further guide the efforts to mitigate the expected damage brought by the projected increase in climate variability. Our results imply that the adaptation to minimize the large KDD impacts in the critical growth stage, such as early planting and development of heat-tolerant genotypes, could be effective adaptation methods to avoid the risk of 2012-like extreme yield loss. Looking into the future, our study implies that modeling of crop yields could be improved by further integrating the process and expert knowledge through, for example, the hybrid model that couples the data-driven deep learning approach and process-based crop models, which could advance the machine intelligence in crop modeling under climate variations.

## Declaration of competing interest

The authors declare that they have no conflicts of interest in this work.

## Appendix Supplementary materials

Image, application 1

## References

[1] FAO (2018). The state of food security and nutrition in the world 2018: building climate resilience for food security and nutrition. Food & Agriculture Org.

[2] Lobell D.B., Roberts M.J., Schlenker W. (2014). Greater sensitivity to drought accompanies maize yield increase in the. U.S. Midwest, Science (80-.)..

[3] Lobell D.B., Bänziger M., Magorokosho C. (2011). Nonlinear heat effects on African maize as evidenced by historical yield trials. Nat. Clim. Chang..

[4] Schlenker W., Roberts M.J. (2009). Nonlinear temperature effects indicate severe damages to U.S. crop yields under climate change. Proc. Natl. Acad. Sci. U. S. A..

[5] Lesk C., Rowhani P., Ramankutty N. (2016). Influence of extreme weather disasters on global crop production. Nature.

[6] Lobell D.B., Hammer G.L., McLean G. (2013). The critical role of extreme heat for maize production in the United States. Nat. Clim. Chang..

[7] Ben-Ari T., Boé J., Ciais P. (2018). Causes and implications of the unforeseen 2016 extreme yield loss in the breadbasket of France. Nat. Commun..

[8] Schewe J., Gosling S.N., Reyer C. (2019). State-of-the-art global models underestimate impacts from climate extremes. Nat. Commun..

[9] Schauberger B., Archontoulis S., Arneth A. (2017). Consistent negative response of US crops to high temperatures in observations and crop models. Nat. Commun..

[10] Lobell D.B., Sibley A., Ivan Ortiz-Monasterio J. (2012). Extreme heat effects on wheat senescence in India. Nat. Clim. Chang..

[11] Wang E., Martre P., Zhao Z. (2017). The uncertainty of crop yield projections is reduced by improved temperature response functions. Nat. Plants..

[12] Pachauri R.K., Allen M.R., Barros V.R. (2014). Climate change 2014: synthesis report. Contribution of Working Groups I, II and III to the fifth assessment report of the Intergovernmental Panel on Climate Change. Ipcc.

[13] Reichstein M., Camps-Valls G., Stevens B. (2019). Deep learning and process understanding for data-driven Earth system science. Nature.

[14] Racah E., Beckham C., Maharaj T. (2017). ExtremeWeather: A large-scale climate dataset for semi-supervised detection, localization, and understanding of extreme weather events. Adv Neural Inf Process Syst.

[15] Liu Y., Racah E., Prabhat (2016). Application of Deep Convolutional Neural Networks for Detecting Extreme Weather in Climate Datasets. arXiv preprint.

[16] Zhao W., Du S. (2016). Learning multiscale and deep representations for classifying remotely sensed imagery. ISPRS J. Photogramm. Remote Sens..

[17] Lecun Y., Bengio Y., Hinton G. (2015). Deep learning. Nature.

[18] Wang A.X., Tran C., Desai N. (2018). Deep Transfer Learning for Crop Yield Prediction with Remote Sensing Data, Proc. 1st ACM SIGCAS Conf. Comput. Sustain. Soc. - COMPASS.

[19] Cao J., Zhang Z., Tao F. (2021). Integrating Multi-Source Data for Rice Yield Prediction across China using Machine Learning and Deep Learning Approaches. Agric. For. Meteorol..

[20] Kang Y., Ozdogan M., Zhu X. (2020). Comparative assessment of environmental variables and machine learning algorithms for maize yield prediction in the US Midwest. Environ. Res. Lett..

[21] Jiang H., Hu H., Zhong R. (2019). A deep learning approach to conflating heterogeneous geospatial data for corn yield estimation: A case study of the US Corn Belt at the county level. Glob. Chang. Biol..

[22] Lin T., Zhong R., Wang Y. (2020). DeepCropNet: a deep spatial-temporal learning framework for county-level corn yield estimation. Environ. Res. Lett..

[23] Schwalbert R.A., Amado T., Corassa G. (2020). Satellite-based soybean yield forecast: Integrating machine learning and weather data for improving crop yield prediction in southern Brazil. Agric. For. Meteorol..

[24] Zhang L., Zhang Z., Luo Y. (2020). Combining optical, fluorescence, thermal satellite, and environmental data to predict county-level maize yield in China using machine learning approaches. Remote Sens.

[25] Tao F., Zhang Z., Xiao D. (2014). Responses of wheat growth and yield to climate change in different climate zones of China, 1981-2009. Agric. For. Meteorol..

[26] Zipper S.C., Qiu J., Kucharik C.J. (2016). Drought effects on US maize and soybean production: Spatiotemporal patterns and historical changes. Environ. Res. Lett..

[27] Leng G., Huang M. (2017). Crop yield response to climate change varies with crop spatial distribution pattern. Sci. Rep..

[28] Carter E.K., Melkonian J., Steinschneider S. (2018). Rainfed maize yield response to management and climate covariability at large spatial scales. Agric. For. Meteorol..

[29] Montavon G., Samek W., Müller K.R. (2018). Methods for interpreting and understanding deep neural networks. Digit. Signal Process. A Rev. J.

[30] USDA-NASS, Quick Stats 2.0. SDA-NASS, Washington, DC. http://www.nass.usda.gov/quickstats/.

[31] Daly C., Halbleib M., Smith J.I. (2008). Physiographically sensitive mapping of climatological temperature and precipitation across the conterminous United States. Int. J. Climatol. a J. R. Meteorol. Soc..

[32] Schaaf C., Wang Z. (2015). MCD43A4: MODIS/Terra+ Aqua BRDF/Albedo Nadir BRDF Adjusted RefDaily L3 Global-500m V006. NASA EOSDIS L. Process. DAAC..

[33] USDA National Agricultural Statistics Service Cropland Data, Published crop-specific data layer (2019).

[34] Soil Survey Staff (2022). The Gridded National Soil Survey Geographic (gNATSGO) Database. United States Department of Agriculture. Natural Resources Conservation Service.

[35] Gorelick N., Hancher M., Dixon M. (2017). Google Earth Engine: Planetary-scale geospatial analysis for everyone. Remote Sens. Environ..

[36] Jain A.K. (2010). Data clustering: 50 years beyond K-means. Pattern Recognit. Lett..

[37] Van der Maaten L., Hinton G. (2008). Visualizing data using t-SNE. J. Mach. Learn. Res..

[38] Schlenker W., Roberts M.J. (2009). Nonlinear temperature effects indicate severe damages to US crop yields under climate change. Proc. Natl. Acad. Sci.

[39] Troy T.J., Kipgen C., Pal I. (2015). The impact of climate extremes and irrigation on US crop yields. Environ. Res. Lett..

[40] Ramirez-villegas J., Challinor A.J., Thornton P.K. (2013). Global crop exposure to critical high temperatures in the reproductive period : historical trends and future projections. Environ. Res. Lett..

[41] Bolaños J., Edmeades G.O. (1996). The importance of the anthesis-silking interval in breeding for drought tolerance in tropical maize. F. Crop. Res..

[42] Wang Y., Sheng D., Zhang P. (2020). High temperature sensitivity of kernel formation in different short periods around silking in maize. Environ. Exp. Bot..

[43] Hsiao J., Swann A.L.S., Kim S.H. (2019). Maize yield under a changing climate: The hidden role of vapor pressure deficit. Agric. For. Meteorol..

[44] Shrestha R.K., Lei P., Shi D. (2021). Response of maize (Zea mays L.) towards vapor pressure deficit. Environ. Exp. Bot..

[45] Jin Z., Zhuang Q., Tan Z. (2016). Do maize models capture the impacts of heat and drought stresses on yield? Using algorithm ensembles to identify successful approaches. Glob. Chang. Biol..

[46] Zipper S.C., Qiu J., Kucharik C.J. (2016). Drought effects on US maize and soybean production: Spatiotemporal patterns and historical changes. Environ. Res. Lett..

[47] Gao Z., Feng H.Y., Liang X.G. (2021). Adjusting the sowing date of spring maize did not mitigate against heat stress in the North China Plain. Agric. For. Meteorol..

