# Supplementary material for: Detect and attribute the extreme maize yield losses based on spatio-temporal deep learning
Source: Fundam Res. 2022 May 16;3(6):951–9. doi: 10.1016/j.fmre.2022.05.006 (PMC11197588; doi:10.1016/j.fmre.2022.05.006)
Supplement: Supplementary file 1 [file mmc1.docx]

**Supplementary Materials**

**S1. Detailed information of the MTL model**

The MTL model estimated county-level maize yield based on climatic factors (i.e., GDD, KDD, VPD, and PRCP), soil properties (AWC, OM, Sand Content, Clay Content, and Silt Content), and remote sensing derived vegetation index (i.e., NDVI). The model employed an ALSTM network for temporal pattern learning from time-series input of climatic factors and NDVI. An ANN was applied to extract the feature for spatial pattern from the soil input. Then, the temporal and spatial features were combined to multiple output layers constructed by multi-task learning. The output layers were region-specific so that the model estimated the yield with the consideration of localized pattern and spatial variability.

The input of the MTL model consisted of two components: temporal dynamical input and spatial static input. The temporal dynamical input was a time sequence $[x_{1}, x_{2}, \ldots, x_{t}]$ and $x_{t}$ was a vector as $[\mathrm{GDD}_{t}, KDD_{t}, VPD_{t}, PRCP_{t}, NDVI_{t}]$ at time step *t*. The spatial static input was a vector $x_{s}$ that consisted of soil properties:$[AWC, OM,C_{\mathrm{sand}}, C_{\mathrm{clay}}, C_{\mathrm{silt}}]$.

The ALSTM network for temporal pattern learning consisted of a three-layer LSTM net and an Attention net. Each LSTM layer was composed of LSTM units for selectively store and transport information from the time sequence based on the gating mechanism. The LSTM unit generated the hidden feature vector $h_{t}$ at time step *t* as follows:

|  | $g_{t}^{f}=\sigma\left( W_{f}\cdot\left[ h_{t-1},x_{t} \right]+b_{f} \right)$ | （1） |
| --- | --- | --- |
|  | $g_{t}^{i}=\sigma\left( W_{i}\cdot\left[ h_{t-1},x_{t} \right]+b_{i} \right)$ | （2） |
|  | $S_{t}=g_{t}^{f}\times S_{t-1}+g_{t}^{i}\times\tanh\left( W_{c}\cdot\left[ h_{t-1},x_{t} \right]+b_{c} \right)$ | （3） |
|  | $g_{t}^{o}=\sigma\left( W_{o}\cdot\left[ h_{t-1},x_{t} \right]+b_{o} \right)$ | （4） |
|  | $h_{t}=g_{t}^{o}\times\tanh\left( S_{t} \right)$ | （5） |

where $W_{f}$, $W_{i}$, $W_{o}$, $W_{c}$, $b_{f}$, $b_{i}$, $b_{o}$ and $b_{c}$ were learnable weight matrices and bias vector parameters of forget gate, input gate, output gate and cell state update, respectively. $S_{t}$ was the cell state vector and represented the information selectively accumulated from time step 1 to time step *t*. $g_{t}^{f}$, $g_{t}^{i}$, and $g_{t}^{o}$ were the vectors generated by these gates. The three vectors consisted of numbers ranging from zero to one, for the operation of filtering information. Zero represented total removal, and one represented total reservation. *h_t_* was the hidden feature vector of the LSTM. $\sigma$ and *tanh* were activation functions.

We used an Attention Net to better aggregate the information from multiple time steps for crop yield estimation. The Attention Net adjusted the contribution of each hidden feature captured by the LSTM net to the output through normalized weight parameters. Then, the final temporal feature vector was generated based on the adjustment. The Attention net in our study was a one-layer fully-connected network with the Softmax function. The final temporal feature vector $h^{T}$was calculated as follows:

|  | $\alpha_{t}=softmax(W_{AT}\cdot h_{t}+b_{AT})$ | (6) |
| --- | --- | --- |
|  | $h^{T}=\alpha_{1}h_{1}+\alpha_{2}h_{2}+\ldots+\alpha_{t}h_{t}$ | (7) |

where $\alpha_{t}$ was the attention value, *softmax* was the activation function to normalize $\alpha_{t}$ so that the sum of all attention value equaled one, *W_AT_* was the weight matrix of the Attention Net, and *b_AT_* was the bias vector.

The ANN network for soil pattern learning was a typical three-layer fully-connected network with the relu function for processing the soil input. The multiple-layer structure enabled the ANN network to extract the hidden feature layer by layer. The final hidden feature vector $h^{S}$ was generated as follows:

|  | $h_{1}^{S}=relu(W_{A}^{1}\cdot x_{S}+b_{A}^{1})$ | (8) |
| --- | --- | --- |
|  | $h_{2}^{S}=relu(W_{A}^{2}\cdot h_{1}^{S}+b_{A}^{2})$ | (9) |
|  | $h^{S}=relu(W_{A}^{3}\cdot h_{2}^{S}+b_{A}^{3})$ | (10) |

where $W_{A}^{1}$, $W_{A}^{2}$, $W_{A}^{3}$, $b_{A}^{1}$, $b_{A}^{2}$, and $b_{A}^{3}$ were learnable weight matrices and bias vectors in each fully-connected layer, $h_{1}^{S}$ and $h_{2}^{S}$ were the hidden feature vectors extracted by the first and second layers, respectively. $relu$ was the non-linear activation function enabling the network to capture the non-linear pattern.

Then, the temporal and soil features were combined as a fused hidden feature vector $h$ ($[h^{T}, h^{S}]$) for the yield estimation. Considering the spatial variability at a large spatial scale, we developed six region-specific output layers based on the MTL and spatial clustering results. The yield was estimated as follows:

|  | $y=W_{r}\cdot h+b_{r}$ | (11) |
| --- | --- | --- |

where *r* was the index of the region, *W_r_* and *b_r_* were learnable weight matrix and bias vector of the responding regional specific output layer for region *r*.


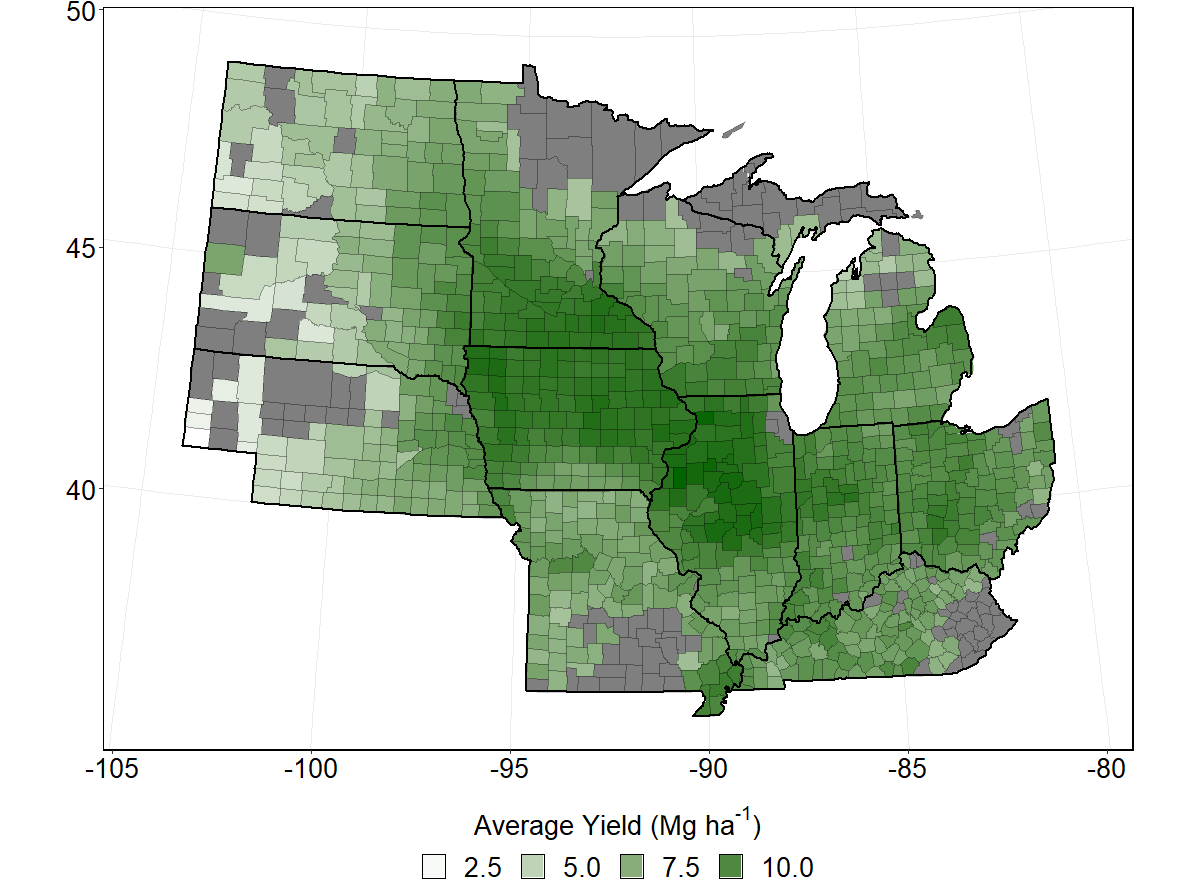


**Fig. S1. County-level average maize yield in the study area from 2006 to 2018.** The counties with grey color are those removed after the data cleaning process.


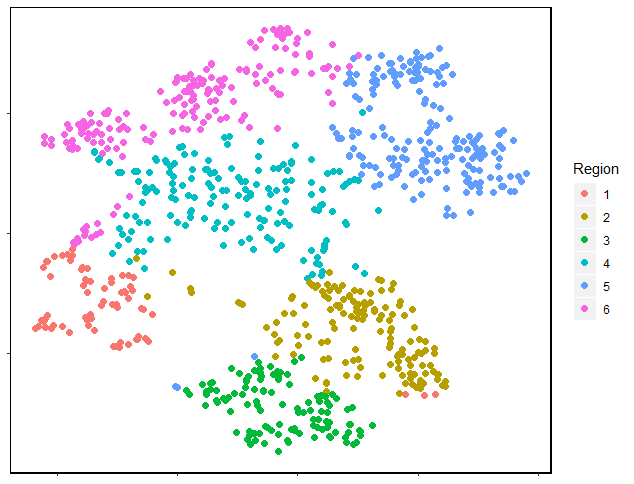


**Fig. S2. Visualization of environmental data by t-distributed stochastic neighbor embedding (t-SNE) for the county-level samples.** The scatter plot showed that the counties were well grouped, which indicates that the clustered regions are well defined based on their complex high-dimension data. The regional distributions of the variables are shown in Fig. S2 (b). Individual axes have no quantitative interpretation due to the nonlinear dimensionality reduction.

**
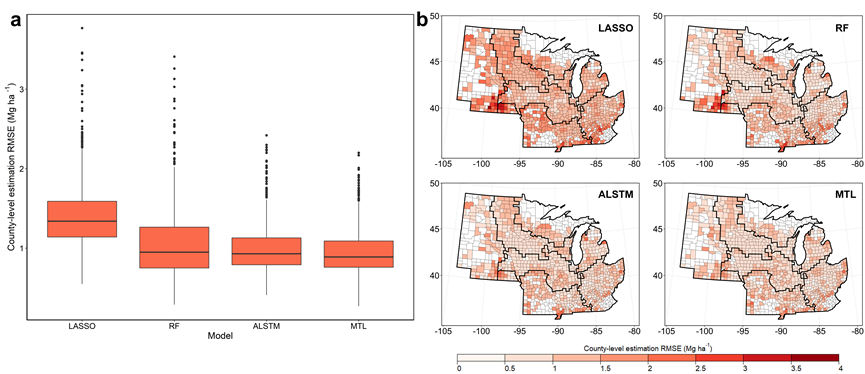
**

**Fig. S3. Performance of four models (LASSO, RF, ALSTM, and MTL) for maize yield estimation at county level in all test years. a**, Boxplots of county-level estimation RMSEs. **b**, Maps of the county-level estimation RMSEs. RMSE indicates the root mean squared error.


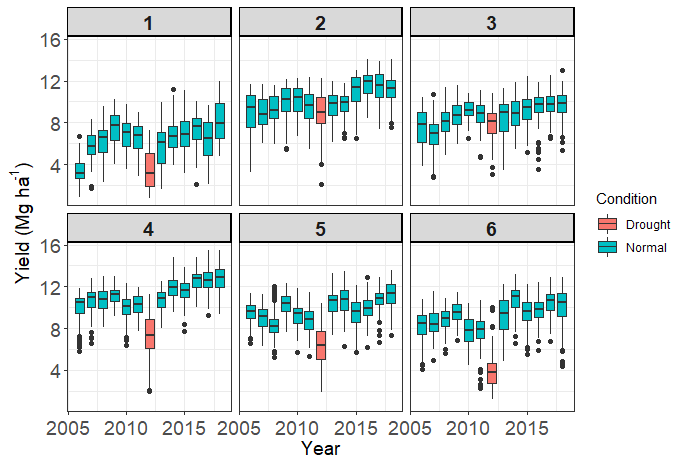


**Fig. S4. Boxplots of county-level maize yield in each region from 2006 to 2018.** The red color represents the year of 2012 when the US Corn Belt suffered heavy heat and drought stresses. The blue color represents the remaining years with normal climatic conditions.


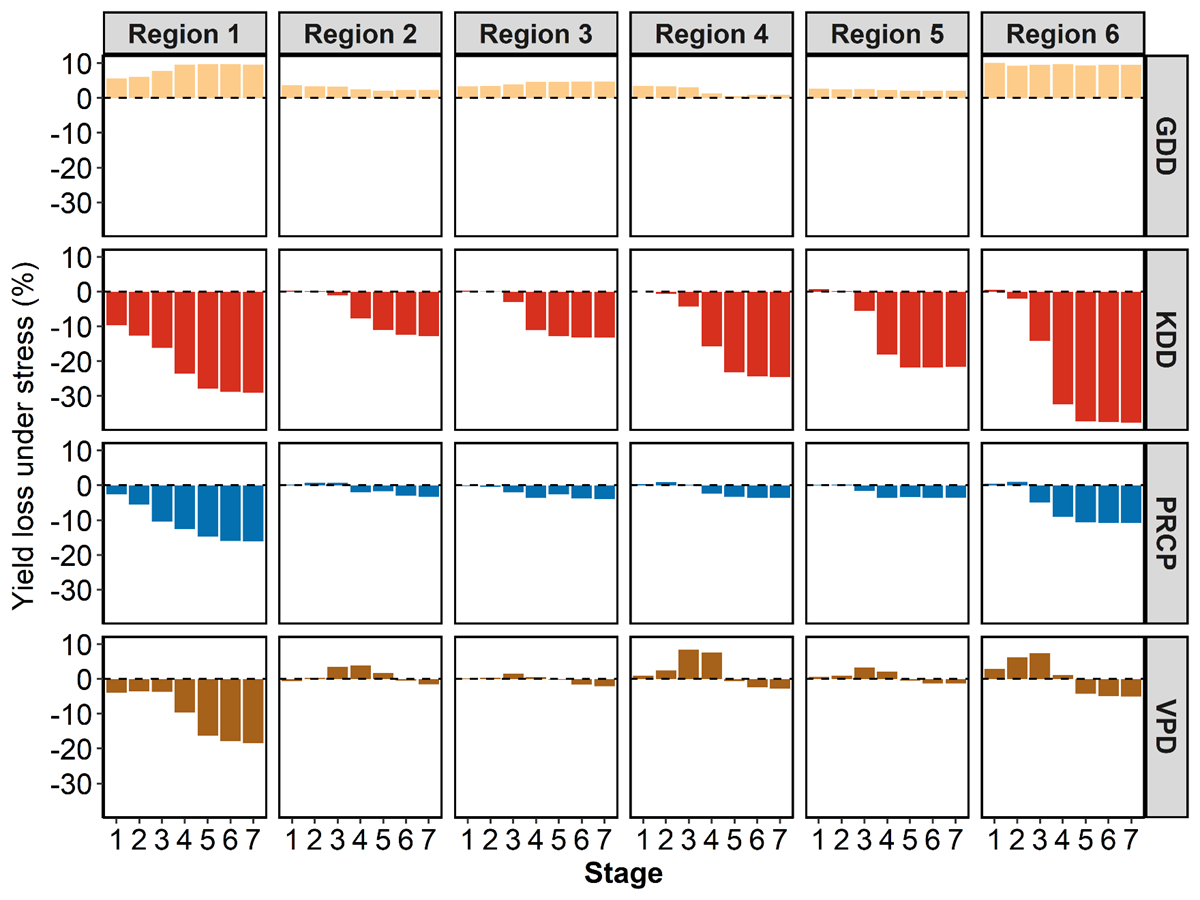


**Fig. S5. The estimated yield loss during the maize growth period across regions in 2012.** The X-axis represents the seven three-week intervals during the whole growth period.


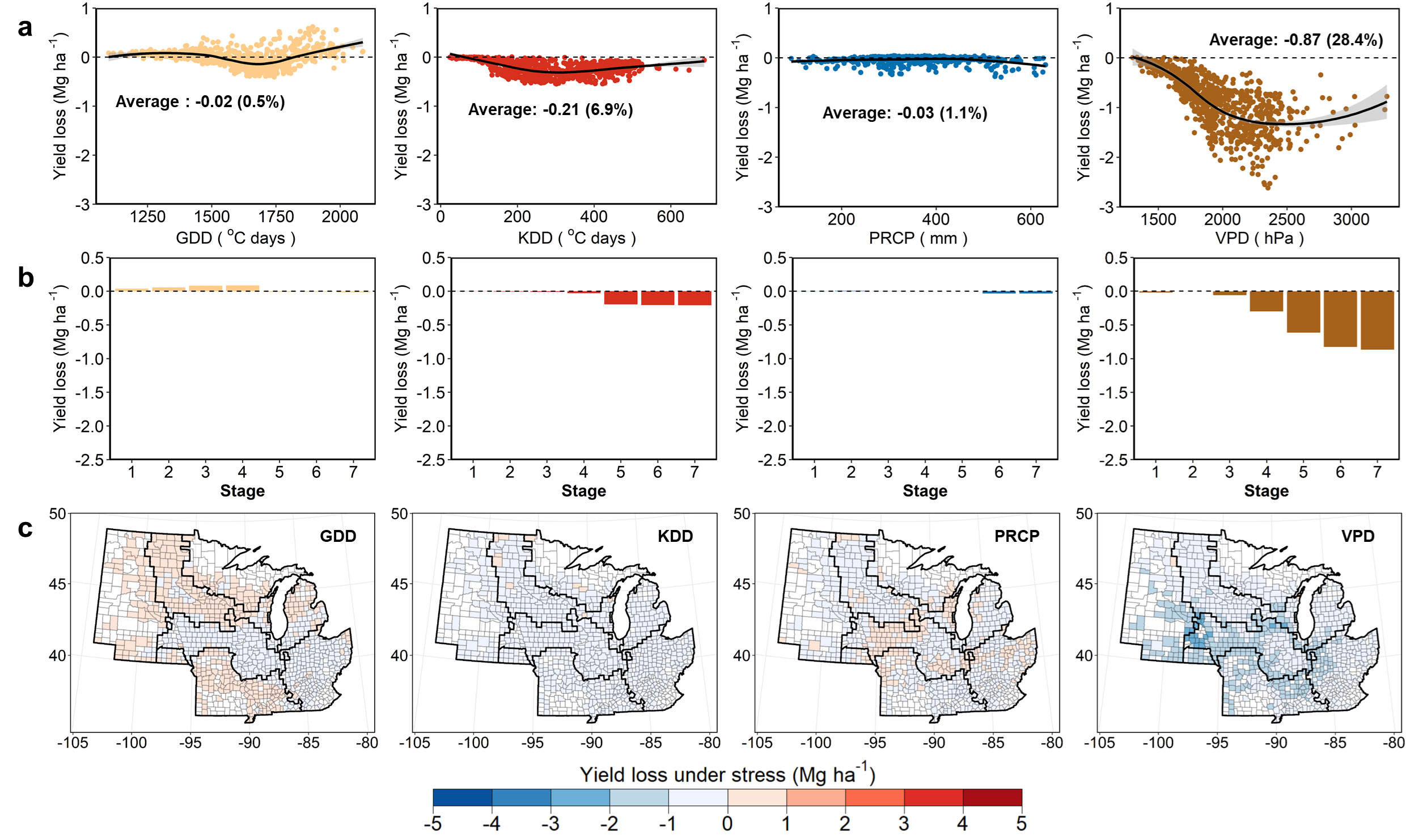


**Fig. S6. Relationship between estimated yield loss by RF and climatic factors and the temporal and spatial patterns of estimated yield loss. a.** The scatterplots between the RF-based estimated yield loss and the corresponding stress in 2012. The yield loss represents the estimated yield reduction causing by the responding climatic stress of 2012 compared to the trend yield in 2012. **b.** The average estimated yield losses as the maize suffered the stresses in 2012 from plant to the responding stage. **c.** The county-level maps of estimated yield loss caused by the corresponding climatic stress in 2012.


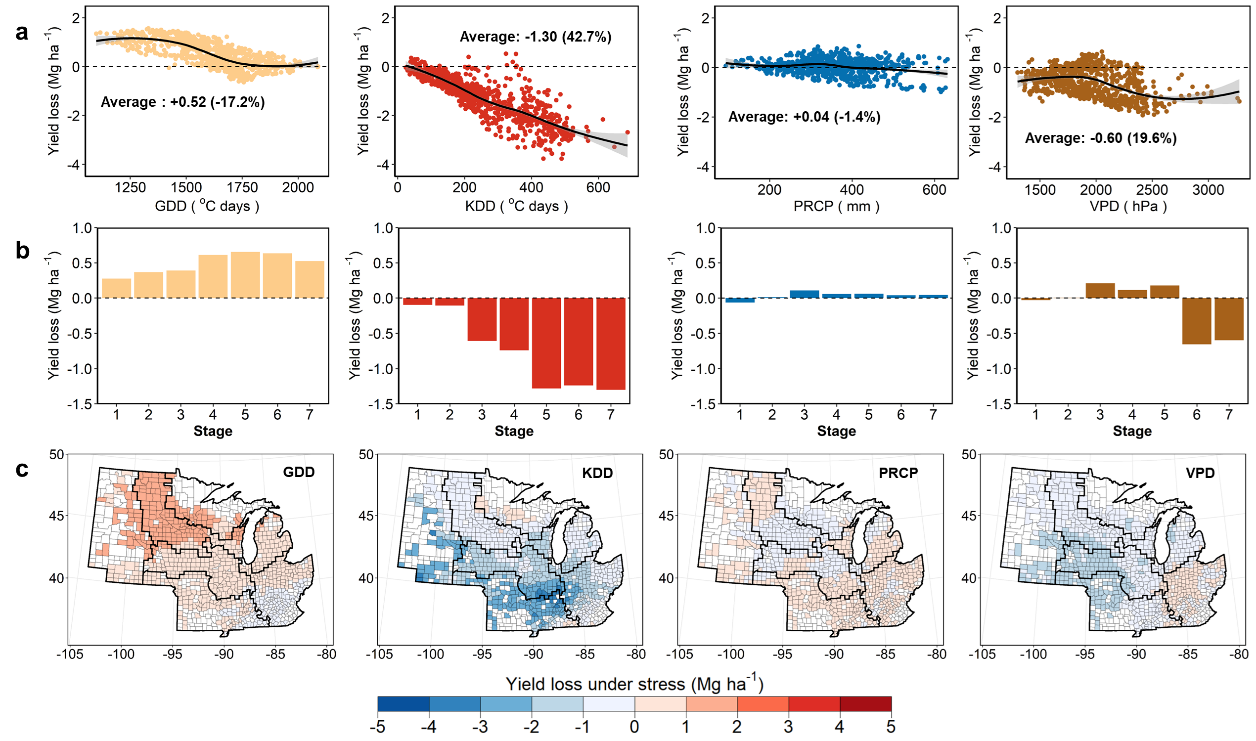


**Fig. S7. Relationship between estimated yield loss by LASSO and climatic** **factors and the temporal and spatial patterns of estimated yield loss. a.** The scatterplots between the LASSO-based estimated yield loss and the corresponding stress in 2012. The yield loss represents the estimated yield reduction causing by the responding climatic stress of 2012 compared to the trend yield in 2012. **b.** The average estimated yield losses as the maize suffered the stresses in 2012 from plant to the responding stage. **c.** The county-level maps of estimated yield loss caused by the corresponding climatic stress in 2012.

Table S1. Paired t-test of yield estimation accuracies among different models

| Comparison | Mean of the RMSE difference (Mg ha^-1^) | | | | | | |
| --- | --- | --- | --- | --- | --- | --- | --- |
|  | US | Region 1 | Region 2 | Region 3 | Region 4 | Region 5 | Region 6 |
| MTL - ALSTM | -0.04* | -0.08* | -0.05 | -0.07* | -0.006 | -0.022 | -0.033 |
| MTL - RF | -0.13** | -0.37** | -0.03 | -0.08* | -0.012 | -0.094 | -0.2* |
| MTL - LASSO | -0.40*** | -0.53** | -0.37*** | -0.27** | -0.23* | -0.31** | -0.54*** |
| ALSTM - RF | -0.09* | -0.29* | 0.02 | -0.003 | -0.006 | -0.07 | -0.16* |
| ALSTM - LASSO | -0.36*** | -0.45* | -0.32** | -0.19* | -0.23 | -0.29** | -0.51*** |
| RF - LASSO | -0.27*** | -0.16 | -0.34** | -0.19** | -0.22* | -0.22** | -0.34** |

* indicates the p-value < 0.05, ** indicates the p-value < 0.01, *** indicates the p-value < 0.001
